# Molecular Mechanisms Linking Diabetes with Increased Risk of Thrombosis

**DOI:** 10.3390/ijms242417465

**Published:** 2023-12-14

**Authors:** Lucy Batten, Thozhukat Sathyapalan, Timothy M. Palmer

**Affiliations:** 1 Biomedical Institute for Multimorbidity, Centre for Biomedicine, Hull York Medical School, University of Hull, Hull HU6 7RX, UK; lucy.batten@hyms.ac.uk; 2 Clinical Sciences Centre, Hull York Medical School, University of Hull, Hull HU6 7RX, UK

**Keywords:** diabetes, thrombosis, platelet, endothelium, cardiovascular disease

## Abstract

This review will provide an overview of what is currently known about mechanisms linking poor glycaemic control with increased thrombotic risk. The leading causes of death in people with diabetes are strokes and cardiovascular disease. Significant morbidity is associated with an increased risk of thrombosis, resulting in myocardial infarction, ischaemic stroke, and peripheral vascular disease, along with the sequelae of these events, including loss of functional ability, heart failure, and amputations. While the increased platelet activity, pro-coagulability, and endothelial dysfunction directly impact this risk, the molecular mechanisms linking poor glycaemic control with increased thrombotic risk remain unclear. This review highlights the complex mechanisms underlying thrombosis prevalence in individuals with diabetes and hyperglycaemia. Post-translational modifications, such as O-GlcNAcylation, play a crucial role in controlling protein function in diabetes. However, the role of O-GlcNAcylation remains poorly understood due to its intricate regulation and the potential involvement of multiple variables. Further research is needed to determine the precise impact of O-GlcNAcylation on specific disease processes.

## 1. Introduction

Diabetes is a chronic metabolic disease characterised by elevated levels of blood glucose due to the body’s impaired ability to produce or respond to the hormone insulin. The number of adults living with diabetes is estimated to be over 420 million, with its prevalence predicted to increase to become the seventh leading cause of global deaths by 2030. The majority of those with a diagnosis of diabetes have type 2 diabetes mellitus (T2DM) [1]. Evidence has shown that the NHS spends around GBP 10 billion a year on diabetes care, equating to around 10% of the entire budget [2]. As the incidence of T2DM is projected to increase significantly over the next 10–15 years, the effective prevention and management of diabetes pose a significant challenge for health and social care systems under increasing pressure from an ageing population in which it is a common co-morbidity [3].

People with diabetes have a 2–4-fold increased risk of developing cardiovascular disease (CVD) and stroke. Approximately 80% of people with diabetes will die of atherothrombotic CVD [4], including major cardiovascular events (MACE), such as acute myocardial infarction (MI) and ischaemic stroke, occurring when platelets aggregate at sites of blood vessel damage to form blood clots, interrupting blood flow and causing tissue hypoperfusion. Poor glycaemic control is known to lead to adverse cardiovascular outcomes, including the increased risk of thrombosis [5].

Multiple mechanisms contribute to the increased risk of thrombosis in diabetes, including activation of coagulation pathways, endothelial dysfunction, and alteration of signalling pathways, leading to an increase in the adhesion, activation, and aggregation of platelets [6]. While high blood glucose can predict susceptibility to CVD, direct mechanisms linking high blood glucose levels associated with poorly controlled diabetes with increased platelet aggregation are unclear.

Hyperglycaemia, a major feature used to determine poor diabetic control, is the main focus of improving clinicians’ management. Rather than a single glucose measurement, the focus has moved to measuring glycated haemoglobin levels (HbA1c), which gives an average blood glucose reading over the life cycle of the red blood cell—usually 8–12 weeks [7]. A person can be diagnosed as having diabetes with an HbA1c level of ≥48 mmol/mol. There is no specific definition of what constitutes “poor control” of diabetes. An HbA1c of >63.9 mmol/mol (8% DCCT, average blood glucose 10.1 mmol/L) is generally used in the USA [8], whereas >53 mmol/mol (7% DCCT, average blood glucose 8.6 mmol/L) is suggested by The National Institute for Health and Care Excellence (NICE) [9]. Targets are based on appropriateness, judged by individual assessment and by weighing up individual risks, particularly of hypoglycaemia.

Antiplatelet medications remain the mainstay of treatment strategies for both primary and secondary prevention of MACE. Despite guidelines detailing when these drugs should be utilised in diabetes, eligible subjects often miss out on these important medications with the potential for devastating results [10]. With regards to surgical intervention for MACE, such as coronary artery bypass surgery, it has been shown in large studies that both short and long-term outcomes are worse in those with diabetes [11].

In this review, we will evaluate mechanisms thought to increase the risk of thrombotic events in diabetes. By recognising new modifications, we may be able to develop targeted medicines to reduce CVD and MACE in people with diabetes whilst minimising side effects.

## 2. Mechanisms Influencing Thrombosis

### 2.1. Endothelial Dysfunction

Endothelial cells, which line the lumen of all blood vessels, act as a cellular monolayer between the blood and underlying vascular smooth muscle cells. The endothelium has a critical role in homeostasis, regulating blood flow by undergoing vasodilation and vasoconstriction, preventing blood loss as well as having pro and anti-inflammatory effects. An imbalance in any of these factors triggers a shift in endothelial cells from a predominantly anti-inflammatory and anti-thrombotic phenotype to a dysfunctional pro-inflammatory and pro-thrombotic one [12].

**Figure 1:**
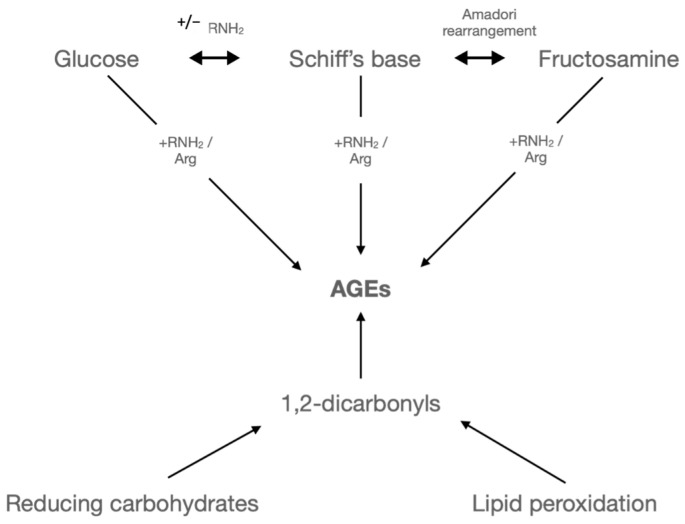
The Maillard reaction. Formation of AGE by the Maillard reaction. It involves a highly intricate process wherein reducing sugars and proteins react under the influence of heat. The Maillard reaction initiates with the interaction of a reducing sugar and an amine, resulting in the formation of glycosylamine. These compounds then undergo Amadori rearrangement to yield a derivative of amino deoxy fructose. The reaction is ongoing, generating highly reactive intermediate substances that ultimately convert glucose molecules to AGE. (Figure adapted from [23]).

Hyperglycaemia causes oxidative stress, resulting in raised levels of glycated proteins and lipids (Figure 1). Advanced glycated end products (AGEs) are proteins or lipids that become glycated and oxidised through a non-enzymatic reaction with glucose or other glycating compounds, such as 3-deoxyglucosone, methylglyoxal, and glyoxal, produced from an increase in fatty acid oxidation [13,14]. This post-translational modification can disrupt the normal functioning of biological processes and is associated with many diseases, including diabetes [15]. Increased levels of AGE and the subsequent modifications of proteins are known to not only play a causative role in cardiovascular complications, such as atherosclerosis and peripheral vascular disease [16], in diabetes but also correlate with the severity of diabetic complications [17].

Damage as a result of AGE modification can occur in multiple ways—by altering the specific function of a protein, modifying the extracellular matrix, resulting in an interaction with matrix receptors on the surface of vascular endothelial cells, and also binding to AGE receptors (RAGE) on macrophages and endothelial cells [14]. RAGE activation results in the activation of multiple transcription pathways, including transcription factor nuclear factor-κB—a universal transcription factor involved in inflammatory and immune responses, which ultimately results in the upregulation of inducible nitric oxide synthase (iNOS) [15,18]. RAGE activation on vascular endothelial cells triggers activation of the coagulation pathway, pro-inflammatory cytokines (such as interleukin-1, interleukin-6, and tumour necrosis factor-alpha), which induce endothelial tissue factor, and the chemokine monocyte chemoattractant protein-1 [19,20].

Medications used to improve blood glucose control improve outcomes in people with diabetes. Metformin works by not only improving glycaemic control by improving the body’s response to glucose but also inhibiting AGE formation in renal tubular cells [21,22].

### 2.2. Oxidative Stress

Oxidative stability is the balance between the rate of free radical formation and elimination and is important in healthy individuals. A decrease in the elimination or an increase in the production results in free radicals and subsequent tissue damage termed oxidative stress [24].

Reactive oxygen species (ROS) are produced during reduction–oxidation reactions in the progression from O_2_ to H_2_O. The superoxide anion (O_2_•−) influences vascular function and serves as a precursor to most other ROS. Superoxide dismutase (SOD) catalyses the conversion of O_2_•− into hydrogen peroxide (H_2_O_2_) by partitioning. Partial reduction of H_2_O_2_ forms hydroxyl radicals (OH•). Catalase and glutathione peroxidase (GPx) fully reduce H_2_O_2_ to H_2_O. Myeloperoxidase (MPO) metabolism of H_2_O_2_ produces a hypochlorous acid [25].

**Figure 2:**
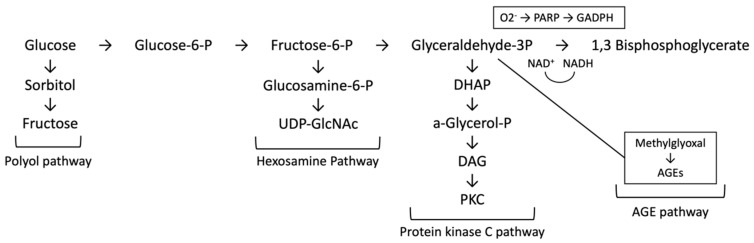
Pathways of hyperglycaemia mediated damage—increased polyol (sorbitol) pathway flux, increased advanced glycation end-product (AGE) formation, activation of protein kinase C (PKC) isoforms, and increased hexosamine pathway flux. (Adapted from [28]).

In diabetes, oxidative stress is thought to be a major contributor to the development of diabetic complications due to the increased production of free radicals in states of hyperglycaemia [26]. The link between hyperglycaemia-induced oxidative stress and complications in diabetes is complex, and four hypotheses have been suggested to explain this: increased polyol (sorbitol) pathway flux, increased AGE formation, activation of protein kinase C (PKC) isoforms, and increased hexosamine pathway flux (Figure 2) [27,28,29].

Glycaemic variability has also been shown to increase morbidity by increasing the risk of diabetic complications, which may not be apparent when looking at overall diabetes control using current methods, such as HbA1c monitoring [30,31]. These intermittent swings of high glucose have been shown to cause an increase in oxidative stress and endothelial dysfunction.

**Figure 3:**
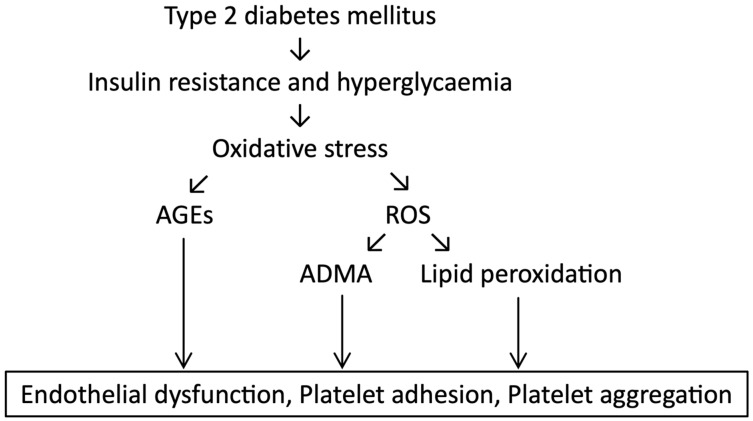
Hyperglycaemia and subsequent effects on the endothelium. The consequences of hyperglycaemia and insulin resistance, as seen in T2DM, on oxidative stress and subsequent endothelial damage. (Figure adapted from [34]).

Overall, glycaemic control seen in poorly controlled diabetes (including hyperglycaemia and glycaemic variability) leads to an increase in ROS and suppression of host antioxidant defence systems (Figure 3) [28].

The disruption in oxidative equilibrium is believed to contribute to nervous degeneration, leading to peripheral diabetic neuropathy [32]. Damage to DNA from oxidative stress prompts excessive activation of the nuclear enzyme poly (ADP-ribose) polymerase-1 (PARP-1). A study conducted by Obrosova et al. (2009) demonstrated that inhibiting PARP alleviated dysfunction and degeneration in small sensory nerve fibres [33].

### 2.3. Platelet Hyperactivity

Platelet hyperactivity is detectable in diabetes well before any vascular changes occur [35]. Platelets isolated from patients with diabetes are known to have higher rates of aggregation than those without diabetes when exposed to low concentrations of pro-thrombotic agonists, such as collagen, ADP, and thrombin [36,37]. The consumption of platelets by aggregation results in an accelerated production of more reactive platelets. This acceleration of platelet turnover subsequently results in higher levels of larger, hyper-reactive, reticulated platelets [38,39]. There is also increased potential for aggregation due to the release from alpha-granules of serotonin and the C-X-C chemokine beta-thromboglobulin and increased production of thromboxaneA2. This lowers the threshold for platelet activation, possibly explaining why there is an increased incidence of thrombosis and vascular dysfunction [40].

Various adhesion molecules are involved in platelet activation and atherosclerosis, and accelerated atherosclerosis is the main underlying factor contributing to the high risk of atherothrombotic events in patients with diabetes mellitus. Specific adhesion molecules are involved in acute platelet activation; CD62P is found on the surfaces of activated endothelial cells and activated platelets and functions as a cell adhesion molecule. CD36, a receptor for thrombospondin-1, functions as a suppressor of angiogenesis [41]. CD31, also known as platelet-endothelial cell adhesion molecule-1, acts as both an adhesive and a signalling protein [42]. They are stored in granules and rapidly mobilised from intracellular membrane stores to the cell surface, where they can then interact with binding partners in acute platelet activation [43,44,45,46]. Elevated levels of platelets CD62P and CD63 have been described in people with type 1 and type 2 diabetes [47,48] and were significantly higher in people with long-term complications of diabetes, such as diabetic nephropathy [46]. The role of CD31 in platelets remains unclear [45], but there has been a hypothesised link between thrombosis and inflammation [44,49]. Other adhesion molecules, such as CD36 and CD49b, are constitutively expressed on the platelet surface, and their expression increases in chronic metabolic dysfunction, as seen in obesity, insulin resistance, and atherosclerosis [50,51,52,53]. Generally, improved control of glycaemia leads to a reduction in the expression of platelet activation markers CD31, CD49b, CD62P, and CD63 in people with T2DM. This beneficial effect may play a role in the strong association between improved metabolic control and reduced development of diabetic complications [48].

Oxidised low-density lipoprotein (OxLDL) influences the function of platelets through mechanisms that go further than direct activation. It has been shown that both aggregated platelets and platelets in whole blood continue to produce ROS in response to OxLDL. Platelets are targeted by ROS as well as being a source, and the role of ROS is essential for regulating activation and aggregation [54]. Studies have shown that OxLDL and its ligation to CD36 increases ROS in platelets. This pathway may result in unwanted platelet activation [55]. CD36 is a membrane glycoprotein expressed on the surface of macrophages, endothelial cells, and platelets. On macrophages, CD36 is involved in the formation of atherosclerotic lesions through its interaction with OxLDL. Macrophages differentiate during atherosclerosis into foam cells. Foam cells formed by LDL-saturated macrophages and subsequent cell death of macrophages occur at the site of fatty streaks, which are present during the early stages of atherosclerotic plaque development. This process causes the progression of atherosclerosis and the development of unstable plaques that are more liable to rupture [56]. Inflammation caused by foam cells causes arterial narrowing, resulting in established vascular disease. It has also been found that CD36 deficiency reduces atherosclerotic lesion formation [57]. Platelet CD36 promotes atherosclerotic inflammatory processes and is involved in further thrombus formation following atherosclerotic plaque rupture [58].

Acute hyperglycaemia also results in increased activation of platelets, with high levels of P-selectin and other soluble markers of platelet activation observed. This may be responsible for the precipitation of acute adverse cardiovascular events, such as MI, and also stresses the importance of effective management of hyperglycaemia in acute vascular events [59,60].

Studies have shown that an improvement in blood sugar control, proven by reduced levels of HbA1c following treatment with oral hypoglycaemic agents and/or insulin, results in a significant decrease in levels of the platelet activation markers CD62P and CD63. There was no change, however, in levels of LDL and HDL, as well as total cholesterol and triglycerides. Baseline assessments showed raised levels of CD31, CD49b, CD62P, and CD63 in the diabetes group when compared to the non-diabetes control [48].

Platelet activation status is a major factor influencing the individual propensity for thrombosis in people with diabetes [61]. In combination with the modification of lifestyle factors, such as smoking and cholesterol levels, the inhibition of platelets with medications such as aspirin remains the mainstay of treatment [62]. The treatment approach may need to be reviewed with the evolving evidence of platelet activation markers.

### 2.4. Coagulation Pathways

Haemostasis is defined as a process that stops bleeding at the site of an injury while maintaining normal blood flow elsewhere in the circulation [63]. It is formed of two pathways, intrinsic and extrinsic, which join at a specific point, leading to the generation of fibrin, which, together with activated platelets, results in clot formation at sites of vascular injury.

**Figure 4:**
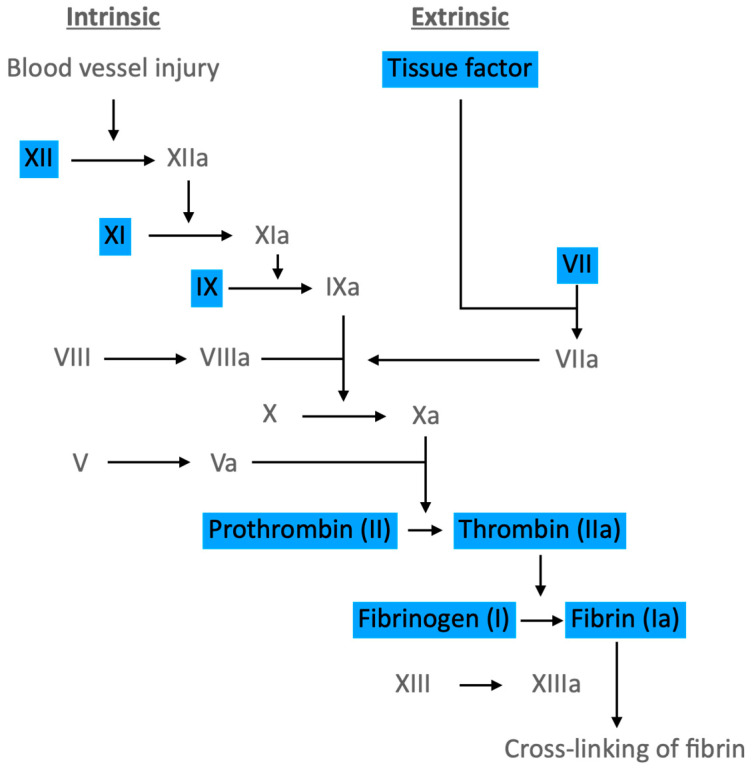
The coagulation pathway. Highlighted proteins increase in hyperglycaemia and insulin-resistant states, contributing to the pro-thrombotic state seen in T2DM. (Figure adapted from [69]).

Haemostasis is reliant on two important processes: primary haemostasis, which is the generation of a platelet “plug” at sites of vascular injury, and secondary haemostasis, which is the deposition of fibrin and subsequent crosslinking by Factor XIIIa formed by the coagulation pathway (Figure 4), which forms a mesh strengthening the platelet plug [64]. *Factor VII and exposed tissue factor bind, forming a complex. This subsequently activates Factor IX and Factor X, which then results in the activation of Factor V. Thrombin also exerts action on Factor VIII, forming a complex with Factor IXa and enhancing the coagulation process [65].*

The fibrinolysis pathway balances this to allow the avoidance of pathological bleeding or thrombosis.

Increased levels of thrombin are seen in people with diabetes but are particularly significant in those with poor glycaemic control [66]. It has been found in a study by Cefalu et al. [67] that in those with poorly controlled diabetes (defined as an HbA1c > 53 mmol/mol in their study), there were very high concentrations of plasminogen activator inhibitor-1 (PAI-1) detected, indicating hypofibrinolysis, leading the authors to conclude that poor glycaemic control is accompanied by significant increases in PAI-1.

Previous identification of increased factor VII levels in those with diabetes, important in the coagulation cascade, led to studies that proved hyperglycaemia, both induced and in those with diabetes, caused an increase in factor VII levels. Maintenance of euglycaemia corrected these levels and further cemented the theory that hyperglycaemia plays a pivotal role in thrombosis formation in hyperglycaemia in diabetes [68].

It seems that hyperinsulinemia contributes to the pro-thrombotic effects of hyperglycaemia. Studies show that hyperinsulinaemia affects thrombotic markers even when euglycaemic but does show an additive effect when both are present, such as in T2DM. Tissue factor procoagulant activity (TF-PCA) levels are raised in those with T2DM and are the initiator of blood coagulation [70]. In a study by Boden et al., it was found that raising insulin levels alone raised TF-PCA by 30%, whereas raising insulin and glucose levels together increased TF-PCA by 80% [71].

### 2.5. Post-Translational Modifications

Diabetes triggers multiple changes that can ultimately impact post-translational modifications (PTMs), which modulate the function and activity of proteins. [72]. Many non-enzymatic PTMs have been connected to the complications of diabetes due to the harmful effects they have on the structure of proteins [73].

Phosphorylation is vital for the proper functioning of pancreatic-β cells, which are responsible for the production of insulin [74]. The phosphorylation of IRS proteins inhibits function and interferes with insulin signalling, ultimately leading to an insulin-resistant state. The subsequent stress that is placed on the β-cells results in dysfunction, resulting in reduced or absent production of insulin [75]. Activation of phosphorylation of IRS-1 by agents such as cytokines, angiotensin II, and cellular stress results in a negative feedback loop on insulin production [76]. Studies have found that protein acetylation proves a new mechanism for insulin secretion [77]. Acetylation can increase the tendency of proteins to become phosphorylated. Enzymes that control the modification of other proteins and proteins that form large complexes are most likely to undergo the process of acetylation [78].

#### O-GlcNAcylation

**Figure 5:**
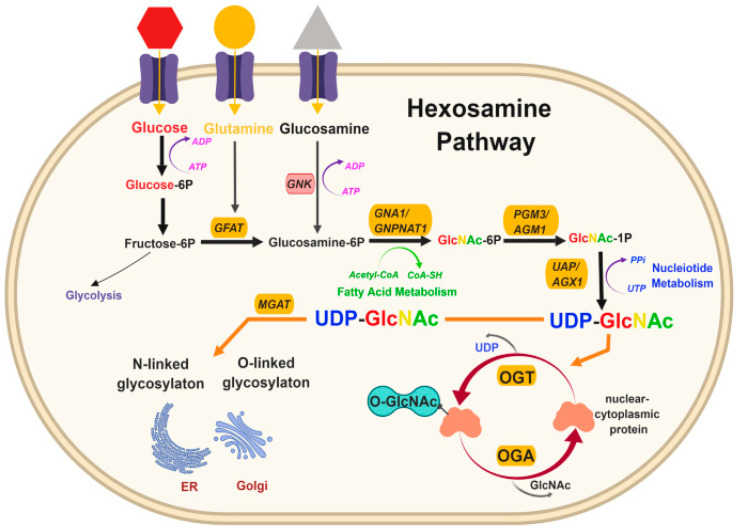
The hexosamine pathway. *The hexosamine pathway is responsible for the O-GlcNAcylation of proteins.* After glucose enters the cell, it is converted, through a series of steps, to UDP-GlcNAc (uridine diphosphate N-acetylglucosamine). GFAT is a key regulator as it catalyses the rate-limiting step in the formation of hexosamine products. UDP-GlcNAc is then used for N-linked and O-linked glycosylation in the endoplasmic reticulum and Golgi apparatus and O-GlcNAc modification of cellular proteins by OGT (O-GlcNAc transferase). (*Figure taken* from [102] under the terms of the *Creative Commons Attribution 4.0 International License*.)

O-GlcNAcylation is a novel PTM that may be responsible for altering critical platelet proteins [7,79]. By changing the function of important platelet proteins, these modifications may increase the risk of inappropriate blood clot formation to trigger heart attack and stroke in people with diabetes. Uncontrolled diabetes results in hyperglycaemia, and it is thought that this causes an increase in the O-GlcNAcylation of specific proteins through the hexosamine biosynthesis pathway (HBP) [80]. This converts up to 5% of cellular glucose to UDP-GlcNAc, a nucleotide sugar for O-GlcNAc transferase (OGT) (Figure 5). It is currently unknown if a clear mechanism exists, but hypothetically, if this were to occur in platelets, it may partly explain why platelets in those with diabetes show increased responsiveness or hyperactivity and, therefore, an increased risk of thrombosis.

For a comprehensive analysis of O-GlcNAcylation, an enrichment step is required to ensure that modified peptides are sufficiently abundant and to ensure they are automatically selected for fragmentation analysis [81]. Many PTMs, including phosphorylation, have site-specific antibodies available for multiple applications. Developing specific antibodies for O-GlcNAc is difficult due to the low immunogenicity of the neutral O-GlcNAc sugar. Two commonly used pan-antibodies for O-GlcNAc are CTD110.6 [82] and RL2 [83]. Lectins have also been used to detect O-GlcNAc. Wheat germ agglutinin (WGA) was first used in 1979 [84], but its lack of specificity for O-GlcNAc has limited its use, although succinylated WGA has been used as it selectively binds to GlcNAc but not to sialic acid, thereby increasing its selectivity versus unmodified WGA [85]. Recombinant lectin PVL produced from *Escherichia coli* has been produced more recently with higher specificity and affinity for proteins with multiple GlcNAc than WGA, as well as *Agrocybe aegerita* lectin 2 and *Psathyrella velutina* lectin [86]. A recent advance has been the development of selective high-affinity substrate traps based on the catalytically inactive mutant of *Clostridium perfringens* NagJ, an orthologue of eukaryotic OGA (termed *Cp*OGA^D298N^). Importantly, *Cp*OGA^D298N^ has been reported to bind O-GlcNAcylated peptides with nanomolar affinity [87] and has been used to enrich O-GlcNAcylated proteins from Drosophila embryos for downstream mass spectrometry analysis and characterisation of the O-GlcNAcome associated with embryonic development [88].

Chemical labelling of O-GlcNAc also allows the identification and quantification of O-GlcNAcylated proteins [89]. Metabolic chemical reporters (MCRs) of glycosylation have been used to label complex glycans, including GlcNAc [90]. The use of 1,3,5,6-tetra-O-acetyl-N-azidoacetyl-glucosamine was able to identify 1500 O-GlcNAcylated proteins, and when used with B-elimination reactions, it was able to identify 185 O-GlcNAcylated sites on 80 proteins [91]. The development of further MCRs has resulted in higher specificity and sensitivity for detecting O-GlcNAcylated proteins, including the creation of MCRs resistant to hydrolysis [92]. Stable Isotope Labelling with Amino Acids in Cell Culture (SILAC) and tandem mass tagging are two approaches that allow quantitative analysis of dynamic changes in protein modifications between different cellular populations [93].

To improve the stability of the O-glycosidic bond, which can result in the loss of identification in collision-induced mass-spectrometry [94], β-elimination followed by the Michael addition with dithiothreitol was developed. This converts the serine and threonine residues of O-GlcNAcylated proteins into more stable derivatives [95], improving the identification of these modified proteins [96]. Mass spectrometry technology has enabled the quantifying and mapping of O-GlcNAc sites with significantly improved accuracy. ETD mass spectrometry is useful to characterise proteins with PTMs and, thus, is a useful tool to detect GlcNAc-modified proteins [97]. The use of high-energy C-trap dissociation (HCD) along with electron transfer dissociation (ETD) complement each other in the identification of O-GlcNAc proteins [98].

O-GlcNAc-regulated pathways have been discovered to play an important role in cell signalling. In a study by Pedowitz et al. [99], it was hypothesised that *O*-GlcNAc levels may contribute to deficits in wound healing. They found that O-GlcNAcylation blocked MYPT1 phosphorylation and cellular contraction and caused downstream dephosphorylation of myosin light chains. Levels of O-GlcNAc also altered the sensitivity of fibroblasts involved during wound healing. This would be important in diabetes, where substrates for O-GlcNAcylation are increased. The balance between MYPT1 and myosin light chain kinase (MLCK) activities is a critical determinant of MLC phosphorylation responsible for platelet contraction important for clot stability [100,101]. Therefore, if a similar phenomenon was found to occur in platelets, there would be significant implications for platelet function.

Endothelial production of nitric oxide (NO) generation by endothelial NO synthase (eNOS) helps to prevent vascular disease by limiting thrombosis and inflammation [103]. It is known that insulin enhances eNOS activity by activating insulin receptor substrates (IRSs), and insulin resistance causes impaired activation of this pathway [104]. Federici et al. [105] studied the effect of O-GlcNAcylation on human coronary artery endothelial cells. They found that the O-GlcNAcylation of IRS and p85 proteins increased with increasing glucose concentration. This supports the idea that hyperglycaemia may contribute to macrovascular complications in diabetes by impairing certain branches of insulin signalling pathways. They also observed that carotid plaques from people with T2DM showed increased levels of O-GlcNAcylated endothelial cells in comparison to those without diabetes by immunostaining with RL2 antibody.

Activation of AKT is important for the calcification of vascular smooth muscle cells through oxidative stress [106,107]. Heath et al. [108] found that activation of AKT by O-GlcNAcylation was key to vascular calcification in diabetes. Chronic hyperglycaemia resulted in increased O-GlcNAcylation, ultimately causing an increase in calcification in vasculature. This study proved that O-GlcNAcylation is an independent contributor to vascular calcification, leading to a higher risk of MACE, and identified O-GlcNAcylation of AKT as a possible therapeutic target in diabetes mellitus.

In a study by Luanpitpong et al. [109], it was discovered that levels of O-GlcNAcylated proteins reduce during the differentiation of megakaryocytic cells, which are responsible for the production of platelets, from hematopoietic stem and progenitor cells. Under healthy conditions, O-GlcNAcylation stabilises the transcription factor c-Myc, a key regulator of cellular metabolism and differentiation, by interfering with its normal process of degradation. Inhibition of O-GlcNAc transferase (OGT) inhibits the O-GlcNAcylation of c-Myc, thus increasing c-Myc degradation and subsequent platelet production. Inhibition of OGT-mediated O-GlcNAcylation may increase platelet production, potentially increasing the risk of thrombosis. As such, this could be a target for future strategies to reduce the risk of thrombosis in those most at risk.

He et al. [110] investigated how hypoglycaemia may affect O-GlcNAcylation alongside phosphorylation rather than hyperglycaemia. They found that glucose deprivation increased O-GlcNAcylaction with glucose deprivation, whereas eNOS phosphorylation was not affected. They found through an analysis of immunoblotting that low glucose levels increased O-GlcNAcylation by 50% through activation of AMP-activated protein kinase (AMPK). In times of low blood glucose, ATP decreases whilst AMP increases. This subsequently leads to activation of the AMPK pathway [111] as AMPK is a sensor of low intracellular ATP levels. As the ATP:AMP ratio decreases, AMPK is activated, and the metabolism moves towards increased catabolism and decreased anabolism [112]. In the liver, AMPK inhibits the production of glucose, cholesterol, and triglycerides and stimulates fatty acid oxidation [113].

Crawford et al. [114] studied the aggregation profiles of platelets obtained from mice. Streptozotocin (STZ) 60 mg/Kg was used to induce hyperglycaemia, mimicking a type 1 model in one group, and leptin-deficient mice, mimicking a type 2 model, were used as a second group. These were both compared to non-hyperglycaemic mice. Their response to low levels of thrombin was measured using aggregometry. Platelets from the STZ-treated mice were hypersensitive and showed aggregation at lower concentrations of agonists compared to the control. Platelets from the leptin-deficient mice showed no differences in reactivity. They found that in platelets treated with O-GlcNAcase and then exposed to variable levels of thrombin, there was an increase in levels of O-GlcNAc. There was not, however, any change to the aggregation profiles compared to untreated platelets. This was backed up by performing the same experiment with banked human platelets, which yielded the same results. Certain alterations of proteins may lead to small, but important, changes in functionality that would not be seen when measuring aggregation alone. This experiment only used exposure to activating stimuli, so it may be possible that responses to inhibitors, such as prostacyclin and NO, are preferentially affected.

## 3. Conclusions

This review proves that the mechanisms involved in the increased prevalence of thrombosis in those with diabetes, and therefore hyperglycaemia, are very complex. Future research should focus on disease-specific mechanisms to identify why there is such a high thrombotic burden in those with diabetes.

Many PTMs have been identified to control protein function and turnover in DM [74]. A particular area of interest would be further research into O-GlcNAcylation. Protein O-GlcNAcylation is tightly regulated with only one enzyme catalysing removal (O-GlcNAse) and one catalysing formation (O-GlcNAc transferase). The lack of evidence describing the role of O-GlcNAcylation is very complex, with the potential for multiple variables contributing to its over- or under-expression. More research is required in this area to determine the exact role O-GlcNAcylation has in specific disease processes.

PTMs are seen in large amounts of proteins involved in diabetes and can affect the stability and, therefore, function of the proteins. There is the possibility for many novel therapeutic targets to be explored in the management of diabetes and the prevention of complications.

## Data Availability

No new data were created or analyzed in this article. Data sharing is not applicable to this article.
